# Methyl 2-(3,5-dinitro­benzamido)-3-methyl­butano­ate

**DOI:** 10.1107/S1600536812040895

**Published:** 2012-10-10

**Authors:** Xiaokun Li, Yuqing Zhao

**Affiliations:** a College of Pharmacy, Henan University of Traditional Chinese Medicine, Zhengzhou, 450008, People’s Republic of China; b School of Civil Engineering and Communication, North China University of Water Source and Electric Power, Zhengzhou 450011, People’s Republic of China

## Abstract

In the title compound, C_13_H_15_N_3_O_7_, the dihedral angle between the amide plane (r.m.s. deviation = 0.008 Å) and the benzene ring is 33.2 (2)°. In the crystal, mol­ecules are connected by N–H⋯O=C hydrogen bonds, forming a chain along the *b*-axis direction.

## Related literature

For the biological activity of related compounds, see: Sykes *et al.* (1999 ▶).
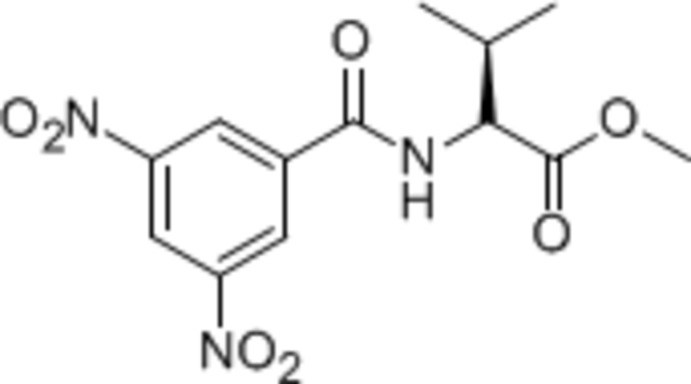


## Experimental

### 

#### Crystal data

C_13_H_15_N_3_O_7_
*M* *_r_* = 325.28
Orthorhombic, 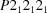

*a* = 7.060 (2) Å
*b* = 9.412 (3) Å
*c* = 23.321 (6) Å
*V* = 1549.8 (7) Å^3^
*Z* = 4
Mo *K*α radiation
μ = 0.12 mm^−1^
*T* = 296 K
0.43 × 0.32 × 0.30 mm

#### Data collection

Bruker APEXII CCD area-detector diffractometer
Absorption correction: multi-scan (*SADABS*; Sheldrick, 1996 ▶) *T*_min_ = 0.660, *T*_max_ = 0.746
9197 measured reflections
3580 independent reflections
3003 reflections with *I* > 2σ(*I*)
*R*_int_ = 0.023

#### Refinement

*R*[*F* ^2^ > 2σ(*F* ^2^)] = 0.045
*wR*(*F* ^2^) = 0.131
*S* = 1.03
3580 reflections
208 parameters
H-atom parameters constrained
Δρ_max_ = 0.20 e Å^−3^
Δρ_min_ = −0.18 e Å^−3^

### 

Data collection: *APEX2* (Bruker, 2007 ▶); cell refinement: *SAINT* (Bruker, 2007 ▶); data reduction: *SAINT*; program(s) used to solve structure: *SHELXTL* (Sheldrick, 2008 ▶); program(s) used to refine structure: *SHELXTL*; molecular graphics: *SHELXTL*; software used to prepare material for publication: *publCIF* (Westrip, 2010 ▶).

## Supplementary Material

Crystal structure: contains datablock(s) I, global. DOI: 10.1107/S1600536812040895/kp2434sup1.cif

Structure factors: contains datablock(s) I. DOI: 10.1107/S1600536812040895/kp2434Isup2.hkl

Additional supplementary materials:  crystallographic information; 3D view; checkCIF report

## Figures and Tables

**Table 1:** Hydrogen-bond geometry (Å, °)

*D*—H⋯*A*	*D*—H	H⋯*A*	*D*⋯*A*	*D*—H⋯*A*
N5—H5*A*⋯O5^i^	0.86	2.12	2.949 (2)	161

Symmetry code: (i) 
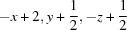
.

**Fig. 1.**
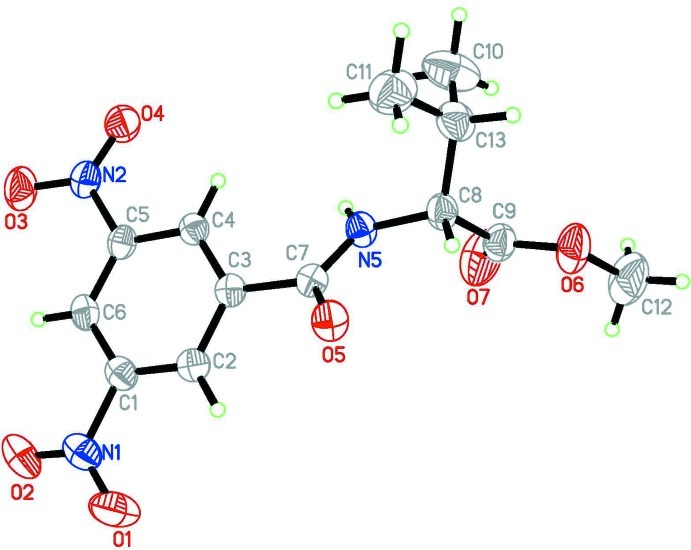
A view of the molecular strcuture of the title compound; the displacement ellipsoids drawn at the 30% probability level.

**Fig. 2.**
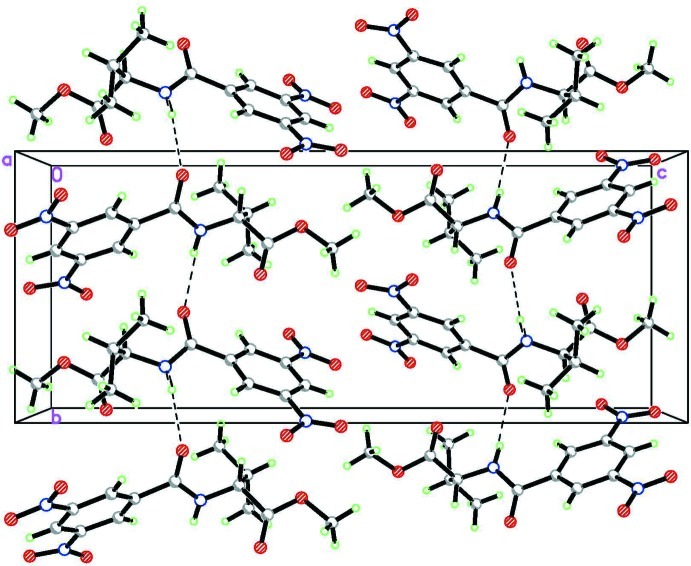
A view of the hydrogen bonded chain running in the [100] direction. The hydrogen bonds are shown as dashed lines.

## supplementary crystallographic information

### Comment

Nitro and ester groups widely exist in variety of biologically active compounds that could be used as prodrugs (Sykes *et al.*, 1999). We synthesized the title compound and determined its crystal structure (Fig. 1) whereas its biological activily is planned to be examined as well. In the crystal of the title compound, the carbonyl group acts as an acceptor and the amide group is a proton donor in the intermolecular N–H···O=C hydrogen bond, forming a chain along the *b* axis (Table 1, Fig. 2).

### Experimental

To a solution of methyl 2-amino-3-methylbutanoate hydrochloride (0.8 g, 5 mmol) and triethylamine (0.5 mL) in dry methylene chloride (100 mL) was added to 3,5-dinitrobenzoyl chloride (1.1 g, 5 mmol)in dry methylene chloride (50 mL) at 273 K. The mixture was allowed to warm to room temperature for 0.5 h. After concentrating, the residue was subjected to chromatography(petroleum ether/ acetone, 4:1) to provide the product as a white crystal (1.2 g, 71.1%).

### Refinement

The C-bound H-atoms were included in calculated positions and treated as riding atoms: C—H = 0.93, 0.96 and 0.98 Å for CH(aromatic), CH_3_ and CH(methine) H-atoms, respectively,and N—H =0.86 Å, with *U*_iso_(H)= k τimes *U*_eq_(parent C-atom, N), where k = 1.5 for CH_3_ H-atoms and k = 1.2 for all other H-atoms. The absolute configuration can be assigned to be R according to the known chirality of the precursor, only.

### Crystal data

**Table:**

C_13_H_15_N_3_O_7_	*F*(000) = 680
*M**_r_* = 325.28	*D*_x_ = 1.394 Mg m^−^^3^
Orthorhombic, *P*2_1_2_1_2_1_	Mo *K*α radiation, λ = 0.71073 Å
Hall symbol: P 2ac 2ab	Cell parameters from 9233 reflections
*a* = 7.060 (2) Å	θ = 1.0–22.7°
*b* = 9.412 (3) Å	µ = 0.12 mm^−^^1^
*c* = 23.321 (6) Å	*T* = 296 K
*V* = 1549.8 (7) Å^3^	Block, colourless
*Z* = 4	0.43 × 0.32 × 0.30 mm

### Data collection

**Table:**

Bruker APEXII CCD area-detector diffractometer	3580 independent reflections
Radiation source: fine-focus sealed tube	3003 reflections with *I* > 2σ(*I*)
Graphite monochromator	*R*_int_ = 0.023
phi and ω scans	θ_max_ = 27.7°, θ_min_ = 1.8°
Absorption correction: multi-scan (*SADABS*; Sheldrick, 1996)	*h* = −7→9
*T*_min_ = 0.660, *T*_max_ = 0.746	*k* = −12→12
9197 measured reflections	*l* = −26→30

### Refinement

**Table:**

Refinement on *F*^2^	Primary atom site location: structure-invariant direct methods
Least-squares matrix: full	Secondary atom site location: difference Fourier map
*R*[*F*^2^ > 2σ(*F*^2^)] = 0.045	Hydrogen site location: inferred from neighbouring sites
*wR*(*F*^2^) = 0.131	H-atom parameters constrained
*S* = 1.03	*w* = 1/[σ^2^(*F*_o_^2^) + (0.0802*P*)^2^ + 0.1123*P*] where *P* = (*F*_o_^2^ + 2*F*_c_^2^)/3
3580 reflections	(Δ/σ)_max_ < 0.001
208 parameters	Δρ_max_ = 0.20 e Å^−^^3^
0 restraints	Δρ_min_ = −0.18 e Å^−^^3^

### Special details

**Table:**

Geometry. All e.s.d.'s (except the e.s.d. in the dihedral angle between two l.s. planes) are estimated using the full covariance matrix. The cell e.s.d.'s are taken into account individually in the estimation of e.s.d.'s in distances, angles and torsion angles; correlations between e.s.d.'s in cell parameters are only used when they are defined by crystal symmetry. An approximate (isotropic) treatment of cell e.s.d.'s is used for estimating e.s.d.'s involving l.s. planes.
Refinement. Refinement of *F*^2^ against ALL reflections. The weighted *R*-factor *wR* and goodness of fit *S* are based on *F*^2^, conventional *R*-factors *R* are based on *F*, with *F* set to zero for negative *F*^2^. The threshold expression of *F*^2^ > σ(*F*^2^) is used only for calculating *R*-factors(gt) *etc*. and is not relevant to the choice of reflections for refinement. *R*-factors based on *F*^2^ are statistically about twice as large as those based on *F*, and *R*- factors based on ALL data will be even larger. C10, C11, C13 and C12 belong to terminal alkyl chains which show signs of disorder and have higher thermal parameters.

### Fractional atomic coordinates and isotropic or equivalent isotropic displacement parameters (Å^2^)

**Table:**

	*x*	*y*	*z*	*U*_iso_*/*U*_eq_	
C1	1.2857 (3)	0.28856 (19)	0.10037 (8)	0.0526 (4)	
C2	1.2504 (3)	0.23672 (19)	0.15455 (8)	0.0503 (4)	
H2A	1.3390	0.1801	0.1732	0.060*	
C3	1.0795 (2)	0.27084 (17)	0.18071 (7)	0.0453 (4)	
C4	0.9487 (3)	0.35376 (18)	0.15216 (8)	0.0469 (4)	
H4A	0.8350	0.3785	0.1696	0.056*	
C5	0.9895 (3)	0.39948 (18)	0.09717 (7)	0.0495 (4)	
C6	1.1576 (3)	0.3692 (2)	0.07014 (8)	0.0521 (4)	
H6A	1.1834	0.4015	0.0333	0.062*	
C7	1.0425 (2)	0.21140 (17)	0.23943 (7)	0.0460 (4)	
C8	0.8869 (3)	0.2398 (2)	0.33103 (8)	0.0557 (4)	
H8A	0.9465	0.1466	0.3358	0.067*	
C9	0.9790 (4)	0.3399 (3)	0.37526 (9)	0.0671 (6)	
C10	0.5694 (5)	0.3567 (4)	0.3394 (2)	0.1160 (12)	
H15A	0.4365	0.3390	0.3442	0.174*	
H15B	0.5902	0.4049	0.3036	0.174*	
H15C	0.6142	0.4149	0.3703	0.174*	
C11	0.5992 (4)	0.1150 (4)	0.29434 (14)	0.0947 (9)	
H14A	0.4656	0.1019	0.2998	0.142*	
H14B	0.6627	0.0253	0.2983	0.142*	
H14C	0.6221	0.1526	0.2567	0.142*	
C12	1.0720 (6)	0.3594 (4)	0.47134 (13)	0.1166 (13)	
H12A	1.0668	0.3069	0.5066	0.175*	
H12B	1.0072	0.4483	0.4760	0.175*	
H12C	1.2018	0.3769	0.4613	0.175*	
C13	0.6744 (4)	0.2186 (3)	0.33924 (11)	0.0735 (6)	
H13A	0.6560	0.1746	0.3769	0.088*	
N1	1.4675 (3)	0.2555 (2)	0.07277 (9)	0.0671 (5)	
N2	0.8475 (3)	0.48375 (18)	0.06597 (7)	0.0613 (4)	
N5	0.9379 (2)	0.29050 (15)	0.27435 (6)	0.0506 (4)	
H5A	0.9000	0.3728	0.2632	0.061*	
O1	1.5804 (3)	0.1847 (3)	0.09906 (10)	0.0990 (6)	
O2	1.4962 (3)	0.3026 (2)	0.02520 (8)	0.0877 (6)	
O3	0.8739 (3)	0.5050 (2)	0.01510 (7)	0.0856 (5)	
O4	0.7125 (3)	0.5274 (2)	0.09269 (7)	0.0781 (5)	
O5	1.1072 (2)	0.09485 (13)	0.25244 (6)	0.0596 (4)	
O6	0.9809 (3)	0.2774 (2)	0.42582 (6)	0.0897 (6)	
O7	1.0395 (5)	0.4534 (3)	0.36573 (10)	0.1163 (9)	

### Atomic displacement parameters (Å^2^)

**Table:**

	*U*^11^	*U*^22^	*U*^33^	*U*^12^	*U*^13^	*U*^23^
C1	0.0540 (10)	0.0489 (9)	0.0547 (10)	−0.0103 (8)	0.0075 (8)	−0.0126 (8)
C2	0.0526 (9)	0.0426 (8)	0.0556 (10)	−0.0011 (7)	−0.0013 (8)	−0.0040 (7)
C3	0.0525 (9)	0.0375 (7)	0.0460 (8)	−0.0054 (7)	−0.0006 (7)	−0.0030 (7)
C4	0.0503 (9)	0.0439 (8)	0.0464 (8)	−0.0035 (7)	−0.0001 (7)	−0.0047 (7)
C5	0.0614 (10)	0.0427 (8)	0.0445 (8)	−0.0027 (8)	−0.0047 (8)	−0.0028 (7)
C6	0.0639 (11)	0.0497 (9)	0.0426 (8)	−0.0102 (8)	0.0046 (8)	−0.0043 (7)
C7	0.0500 (9)	0.0402 (8)	0.0477 (8)	−0.0039 (7)	−0.0024 (7)	0.0004 (7)
C8	0.0658 (11)	0.0549 (10)	0.0463 (9)	0.0091 (9)	0.0023 (8)	0.0045 (8)
C9	0.0770 (14)	0.0720 (13)	0.0523 (11)	0.0169 (12)	−0.0047 (10)	−0.0061 (9)
C10	0.0821 (19)	0.094 (2)	0.172 (4)	0.0177 (17)	0.034 (2)	0.017 (2)
C11	0.0834 (18)	0.099 (2)	0.102 (2)	−0.0206 (17)	−0.0107 (15)	0.0137 (17)
C12	0.152 (3)	0.130 (3)	0.0680 (16)	0.019 (3)	−0.0295 (19)	−0.0348 (17)
C13	0.0736 (14)	0.0762 (14)	0.0707 (13)	−0.0033 (12)	0.0113 (11)	0.0167 (11)
N1	0.0613 (10)	0.0632 (10)	0.0769 (12)	−0.0070 (9)	0.0174 (9)	−0.0107 (9)
N2	0.0743 (11)	0.0594 (9)	0.0504 (9)	0.0028 (9)	−0.0069 (8)	0.0018 (7)
N5	0.0629 (9)	0.0432 (7)	0.0456 (7)	0.0071 (7)	0.0047 (6)	0.0055 (6)
O1	0.0641 (10)	0.1110 (14)	0.1218 (16)	0.0183 (11)	0.0206 (10)	0.0107 (13)
O2	0.0948 (13)	0.0910 (12)	0.0773 (11)	−0.0046 (11)	0.0372 (10)	−0.0117 (9)
O3	0.1103 (15)	0.0954 (12)	0.0511 (8)	0.0146 (12)	−0.0049 (9)	0.0146 (8)
O4	0.0800 (11)	0.0881 (11)	0.0661 (9)	0.0258 (9)	−0.0040 (8)	0.0041 (8)
O5	0.0776 (9)	0.0431 (6)	0.0580 (7)	0.0098 (6)	0.0016 (7)	0.0035 (6)
O6	0.1274 (15)	0.0904 (11)	0.0512 (8)	0.0167 (12)	−0.0109 (9)	−0.0112 (8)
O7	0.162 (2)	0.0923 (13)	0.0946 (14)	−0.0369 (15)	−0.0343 (14)	−0.0015 (12)

### Geometric parameters (Å, º)

**Table:**

C1—C6	1.375 (3)	C9—O6	1.318 (3)
C1—C2	1.377 (3)	C10—C13	1.497 (4)
C1—N1	1.469 (3)	C10—H15A	0.9600
C2—C3	1.390 (3)	C10—H15B	0.9600
C2—H2A	0.9300	C10—H15C	0.9600
C3—C4	1.380 (3)	C11—C13	1.526 (4)
C3—C7	1.502 (2)	C11—H14A	0.9600
C4—C5	1.383 (3)	C11—H14B	0.9600
C4—H4A	0.9300	C11—H14C	0.9600
C5—C6	1.373 (3)	C12—O6	1.462 (3)
C5—N2	1.471 (3)	C12—H12A	0.9600
C6—H6A	0.9300	C12—H12B	0.9600
C7—O5	1.226 (2)	C12—H12C	0.9600
C7—N5	1.328 (2)	C13—H13A	0.9800
C8—N5	1.451 (2)	N1—O1	1.206 (3)
C8—C13	1.525 (3)	N1—O2	1.212 (3)
C8—C9	1.541 (3)	N2—O4	1.211 (2)
C8—H8A	0.9800	N2—O3	1.217 (2)
C9—O7	1.171 (3)	N5—H5A	0.8600
			
C6—C1—C2	123.15 (18)	H15A—C10—H15B	109.5
C6—C1—N1	117.83 (18)	C13—C10—H15C	109.5
C2—C1—N1	119.02 (19)	H15A—C10—H15C	109.5
C1—C2—C3	118.55 (18)	H15B—C10—H15C	109.5
C1—C2—H2A	120.7	C13—C11—H14A	109.5
C3—C2—H2A	120.7	C13—C11—H14B	109.5
C4—C3—C2	119.99 (17)	H14A—C11—H14B	109.5
C4—C3—C7	122.29 (16)	C13—C11—H14C	109.5
C2—C3—C7	117.70 (16)	H14A—C11—H14C	109.5
C3—C4—C5	118.94 (17)	H14B—C11—H14C	109.5
C3—C4—H4A	120.5	O6—C12—H12A	109.5
C5—C4—H4A	120.5	O6—C12—H12B	109.5
C6—C5—C4	122.75 (17)	H12A—C12—H12B	109.5
C6—C5—N2	118.28 (16)	O6—C12—H12C	109.5
C4—C5—N2	118.97 (17)	H12A—C12—H12C	109.5
C5—C6—C1	116.59 (17)	H12B—C12—H12C	109.5
C5—C6—H6A	121.7	C10—C13—C8	111.9 (2)
C1—C6—H6A	121.7	C10—C13—C11	112.6 (3)
O5—C7—N5	123.84 (16)	C8—C13—C11	109.9 (2)
O5—C7—C3	119.61 (16)	C10—C13—H13A	107.4
N5—C7—C3	116.54 (14)	C8—C13—H13A	107.4
N5—C8—C13	113.70 (18)	C11—C13—H13A	107.4
N5—C8—C9	107.70 (17)	O1—N1—O2	123.8 (2)
C13—C8—C9	114.29 (18)	O1—N1—C1	118.12 (19)
N5—C8—H8A	106.9	O2—N1—C1	118.0 (2)
C13—C8—H8A	106.9	O4—N2—O3	124.5 (2)
C9—C8—H8A	106.9	O4—N2—C5	117.71 (17)
O7—C9—O6	125.0 (2)	O3—N2—C5	117.81 (19)
O7—C9—C8	125.7 (2)	C7—N5—C8	120.85 (15)
O6—C9—C8	109.3 (2)	C7—N5—H5A	119.6
C13—C10—H15A	109.5	C8—N5—H5A	119.6
C13—C10—H15B	109.5	C9—O6—C12	114.7 (3)

### Hydrogen-bond geometry (Å, º)

**Table:**

*D*—H···*A*	*D*—H	H···*A*	*D*···*A*	*D*—H···*A*
N5—H5*A*···O5^i^	0.86	2.12	2.949 (2)	161

Symmetry code: (i) −*x*+2, *y*+1/2, −*z*+1/2.
